# Increases in Theta Oscillatory Activity During Episodic Memory Retrieval Following Mindfulness Meditation Training

**DOI:** 10.3389/fnhum.2019.00311

**Published:** 2019-09-04

**Authors:** Erika Nyhus, William Andrew Engel, Tomas Donatelli Pitfield, Isabella Marie Wang Vakkur

**Affiliations:** ^1^Department of Psychology, Bowdoin College, Brunswick, ME, United States; ^2^Program in Neuroscience, Bowdoin College, Brunswick, ME, United States

**Keywords:** mindfulness meditation, episodic memory, memory retrieval, EEG, theta oscillations

## Abstract

Mindfulness meditation has been shown to improve episodic memory and increase theta oscillations which are known to play a role in episodic memory retrieval. The present study examined the effect of mindfulness meditation on episodic memory retrieval and theta oscillations. Using a longitudinal design, subjects in the mindfulness meditation experimental group who underwent 4 weeks of mindfulness meditation training and practice were compared to a waitlist control group. During the pre-training and post-training experimental sessions, subjects completed the Five Facet Mindfulness Questionnaire (FFMQ) and studied adjectives and either imagined a scene (Place Task) or judged its pleasantness (Pleasant Task). During the recognition test, subjects decided which task was performed with each word (“Old Place Task” or “Old Pleasant Task”) or “New.” FFMQ scores and source discrimination were greater post-training than pre-training in the mindfulness meditation experimental group. Electroencephalography (EEG) results revealed that for the mindfulness meditation experimental group theta power was greater post-training than pre-training in right frontal and left parietal channels and changes in FFMQ scores correlated with changes in theta oscillations in right frontal channels (*n* = 20). The present results suggest that mindfulness meditation increases source memory retrieval and theta oscillations in a fronto-parietal network.

## Introduction

Mindfulness meditation has been defined as the practice of becoming aware of present-moment experience with a compassionate, non-judgmental stance (Kabat-Zinn, 1990), but this definition is not universally accepted nor is there agreement on the what constitutes mindfulness. Although there are other definitions of mindfulness that focus on the formal practice of sitting meditation, most researchers agree that it is related to attention (reviewed in Van Dam et al., 2017). In recent years, mindfulness meditation has gained popularity and has been used widely by psychologists and educators. Mindfulness meditation has been shown to have positive effects on a number of psychological and health outcomes such as reducing stress, depression, generalized anxiety disorder, addictions, attention deficit disorder, and pain disorders (reviewed in Cahn and Polich, 2006; Holzel et al., 2011b; Tang et al., 2015; Creswell, 2017; Van Dam et al., 2017). Although mindfulness meditation has been shown to have positive effects, these effects are often small and it is not clear how they compare to other interventions (Eberth and Sedlmeier, 2012; Sedlmeier et al., 2012; Goyal et al., 2014). For example, MacCoon et al. (2012) compared mindfulness meditation to an active exercise control group and found that both groups showed equal improvement in psychological outcome measures over time. In addition, recent research has begun to explore the effects of mindfulness meditation on cognition and its neural correlates.

Mindfulness meditation has been shown to improve attention and executive function (reviewed in Lutz et al., 2008; Chiesa et al., 2011; Holzel et al., 2011b; Eberth and Sedlmeier, 2012; Sedlmeier et al., 2012; Tang et al., 2015; Creswell, 2017). For example, a brief, 4-day, mindfulness meditation training improved performance on attention and executive function tasks (symbol digit modality, verbal fluency, and n-back) (Zeidan et al., 2010) and a 2-week mindfulness course improved performance on a working memory task (operation span task) (Mrazek et al., 2013). Again, these effects are often small and it is not clear how they compare to other interventions (Eberth and Sedlmeier, 2012; Sedlmeier et al., 2012; Goyal et al., 2014; MacCoon et al., 2014). Although many studies have shown effects of mindfulness meditation on attention and executive function which should contribute to better episodic memory, less is known about the effects of mindfulness meditation on episodic memory (reviewed in Levi and Rosenstreich, 2018). Recent research has begun to look at the behavioral effects of mindfulness meditation on episodic memory. Meditation training has been shown to increase recognition memory, especially recollection (Brown et al., 2016; Basso et al., 2019) and free recall (Lykins and Baer, 2012).

Episodic memory retrieval involves the interaction of frontal, parietal, and medial temporal lobe regions (Spaniol et al., 2009). Recent research has begun to examine how these distributed regions coordinate activity. Neural oscillations play an important role in communication among neurons within a network. Neural oscillations in different frequencies have been studied extensively in humans and animals and have been shown to be important for episodic memory. Multiple electroencephalography (EEG) studies have shown positive theta (4–8 Hz) effects during episodic memory encoding and retrieval. Many studies have shown greater theta power for subsequently remembered than forgotten items as well as greater theta power for correctly remembered items than new items (reviewed in Nyhus and Curran, 2010). In addition, theta oscillations over frontal electrodes have been shown to be greater under conditions requiring control of episodic retrieval including the retrieval of source information (reviewed in Nyhus and Curran, 2010). Based on this evidence, it has been proposed that theta oscillations allow for top-down control in episodic memory (Klimesch, 1996, 1999; Kahana et al., 2001; Klimesch et al., 2008, 2010; Sauseng et al., 2010), which is consistent with the role of theta oscillations more generally in large-scale brain network dynamics in prefrontal networks supporting cognitive control (reviewed in Cavanagh and Frank, 2014).

Mindfulness meditation has been related to structural and functional differences in brain networks related to episodic memory (Tomasino et al., 2012; Fox et al., 2014, 2016). For example, compared to control subjects, long-term meditators showed increased gray matter volume in prefrontal cortex (Lazar et al., 2005; Luders et al., 2009, 2013b; Kang et al., 2013) and hippocampus (Holzel et al., 2008; Luders et al., 2009, 2013a,b) and the duration of meditation correlated with gray matter density in the prefrontal cortex (Lazar et al., 2005) and the hippocampus (Luders et al., 2013a). Long-term meditators also showed increased connectivity in the temporal component of the superior longitudinal fasciculus (Luders et al., 2011). In a longitudinal controlled study, mindfulness meditation training led to increased hippocampal volume (Holzel et al., 2011a; Luders et al., 2013a). In addition, meditative states and traits are related to activity in prefrontal cortex (Lazar et al., 2000; reviewed in Cahn and Polich, 2006; Sperduti et al., 2012; reviewed in Tang et al., 2012; reviewed in Zeidan, 2015; Tomasino and Fabbro, 2016) and hippocampus (Lou et al., 1999; Lazar et al., 2000; Engstrom et al., 2010). Importantly, meditative states and traits are related to increases in theta power and coherence in both long-term meditators and following mindfulness meditation training (reviewed in Delmonte, 1984; Lou et al., 1999; Kubota et al., 2001; Aftanas and Golosheikin, 2003; reviewed in Cahn and Polich, 2006; Tang et al., 2009; reviewed in Fell et al., 2010; reviewed in Lomas et al., 2015; Brandmeyer and Delorme, 2018; reviewed in Lee et al., 2018). For example, compared to non-expert meditators, expert meditators showed greater theta power during self-reported meditation than mind wandering (Brandmeyer and Delorme, 2018).

Therefore, previous studies have shown that mindfulness meditation increases episodic memory (reviewed in Lykins and Baer, 2012; Brown et al., 2016; Levi and Rosenstreich, 2018; Basso et al., 2019) and theta oscillations (reviewed in Delmonte, 1984; Lou et al., 1999; Kubota et al., 2001; Aftanas and Golosheikin, 2003; reviewed in Cahn and Polich, 2006; Tang et al., 2009; reviewed in Fell et al., 2010; reviewed in Lomas et al., 2015; Brandmeyer and Delorme, 2018; reviewed in Lee et al., 2018). But no study has trained participants in mindfulness meditation and measured theta oscillatory effects during episodic memory. The purpose of the present study was to examine the effect of mindfulness meditation on recollection of specific information from the study episode and theta oscillations. We predicted that mindfulness, source memory, and theta oscillations would increase from pre-training to post-training for a mindfulness meditation group, but not a waitlist control group. Given the recent focus on mindfulness meditation and its effects on cognition and mood, it is important to study the effects of mindfulness meditation using robust and unbiased research (Tang et al., 2015; Creswell, 2017; Van Dam et al., 2017). By using a longitudinal design with matched mindfulness meditation and waitlist control groups and measuring EEG we can gain insight into the neural processes affected by mindfulness meditation during episodic memory retrieval.

## Materials and Methods

### Subjects

Subjects were recruited from the Bowdoin College community through flyers and advertisements in student email lists. Fifty-one people participated in the experiment for payment ($15/hour). All subjects gave informed consent. Data from 11 subjects were discarded because of failure to complete all experimental sessions (*n* = 9), excessive number of bad channels (*n* = 1), and experimenter error (*n* = 1). Of the 40 subjects analyzed, there were 10 male and 10 female subjects ranging from 18 to 22 years old in the mindfulness meditation experimental group and 7 male and 13 female subjects ranging from 18 to 22 years old in the waitlist control group. All subjects were right-handed and fluent English speakers. Subjects participated in two experimental sessions: (1) a study session and test session (2) after 48 to 154 days a study and test session following either mindfulness meditation training or waitlist. The timing of the post-training experimental session was affected by the semester break and, for the mindfulness meditation experimental group, by the 4 weeks of mindfulness meditation training. The post-training experimental session for the mindfulness meditation experimental group occurred 2 to 18 days following the completion of the 4 weeks of mindfulness meditation training. The average time between pre-training and post-training experimental sessions was equal for the mindfulness meditation experimental and waitlist control groups. Subjects were randomly assigned by the experimenters to the mindfulness meditation experimental or waitlist control group. All subjects were meditation naïve. All procedures were approved by the Institutional Review Board of Bowdoin College, in accord with federal guidelines for the protection of human subjects.

### Mindfulness Questionnaire

Prior to each experimental session, subjects were asked to complete the Five Facet Mindfulness Questionnaire (FFMQ) (see Supplementary Material) which is based on a factor analysis of five mindfulness questionnaires (Baer et al., 2006). The five facets of mindfulness are observing (Observe), describing (Describe), acting with awareness (Awareness), non-judging of inner experience (Non-judge), and non-reactivity to inner experience (Non-reactive).

### Episodic Memory Task

#### Stimuli

Experimental stimuli consisted of 800 adjectives (e.g., dirty, happy). 15 additional adjectives were used for practice. The words were common English adjectives roughly equated for word frequency (*M* = 34.86, *SD* = 86.96, range 0:1171) according to the Kucera and Francis (1967) word norms. All adjectives were presented in upper case in white on an LCD computer monitor on a black background subtending a visual angle of approximately 3.7°.

#### Design

Memory status (old, new) and encoding task (place, pleasantness) were manipulated within subjects. Word lists were randomized across encoding task. In each experimental session, subjects were presented with both encoding tasks randomly intermixed. Test key assignments were counterbalanced across subjects.

#### Procedure

In each experimental session, subjects were given instructions and then presented with a short practice study block. Practice study blocks consisted of 10 study words. After completing the practice study block, subjects began the study block.

**FIGURE 1:**
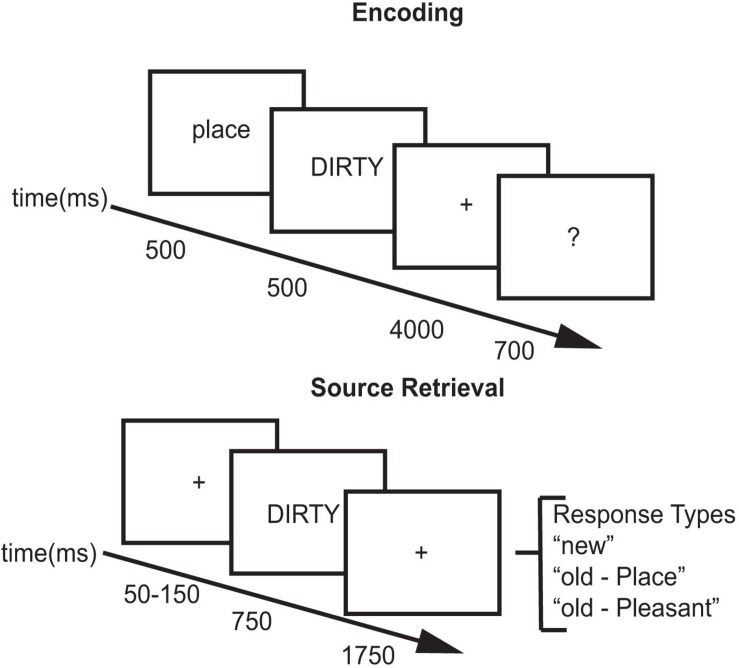
Experimental paradigm.

For each study block, subjects viewed 204 words. Two words at the beginning and two words at the end of the list acted as primacy and recency buffers. For half of the trials, the cue “Place” preceded the word and subjects created a mental image of a spatial scene described by the adjective (place task – e.g., for “DIRTY,” the subject might imagine a messy room). For the other half of the trials, the cue “Pleasant” preceded the word and subjects thought about the meaning of the word and rated its pleasantness (pleasantness task – e.g., for “HAPPY,” the subject might think that the word was pleasant, see Figure 1). After performing the encoding task for each word, subjects were asked to rate how successfully they performed each encoding task. Using their right hand, subjects pressed one of four buttons: (1) unsuccessful; (2) partially; (3) with effort; (4) with ease. Each word was preceded by a 500 ms cue (Place/Pleasant) indicating which encoding task to perform followed by a 200 ms blank screen. The adjective was then presented for 500 ms followed by a 4000 ms fixation during which they performed the encoding task. The fixation cross then changed to a question mark for 700 ms during which the subjects made their response (see Figure 1).

After the study block, subjects were fitted with the EEG electrode cap (see EEG procedures below) and then performed a short practice test block. Practice test blocks consisted of 15 test words. After completing the practice test block, subjects began the test blocks. The interval between the end of the study list and beginning of the test list was approximately 30 min.

Across all test blocks, subjects viewed 480 words: 80 buffers, 200 studied words, and 200 new words. Two words at the beginning and two words at the end of each block acted as movement buffers as subjects tend to move at the beginning and end of each block. Test sessions contained the 200 studied words intermixed with 200 new words. Subjects were tested in blocks of 20 words with a self-timed break in between the blocks. Test trials included a variable duration (50–150 ms) green fixation cross (+) followed by a test word. For each test word, participants decided if the word was new, or if they recognized the word, which task they had performed with it. Each test word was presented for 750 ms followed by a red fixation (+) for 1750 ms. Upon appearance of the test word, subjects could provide a response using a button box. Using their index, middle, and ring finger of their right hand, subjects pressed one key for New, one key for Place, and one key for Pleasant (see Figure 1). Responses made after display offset were counted as non-response trials. Subjects took approximately 2 h to complete the study/test block in each experimental session.

#### EEG Recording and Analysis

During the testing phase of the experiment, scalp voltages were collected with a 64-channel actiCHamp system (Brain Products, Munich, Germany). Amplified analog voltages (0.1–100 Hz bandpass) were digitized at 500 Hz. Individual sensors were adjusted until impedances were less than 25 kΩ.

EEG was further preprocessed using EEGLab (Delorme and Makeig, 2004). The EEG was downsampled to 250 Hz and digitally high-pass filtered at 1 Hz and low-pass filtered at 100 Hz. Individual channels were replaced on a trial-by-trial basis with a spherical spline algorithm (Srinivasan et al., 1996). EEG was measured with respect to a frontal reference (Fz), but an average-reference transformation was used to minimize the effects of reference-site activity and accurately estimate the scalp topography of the measured electrical fields (Dien, 1998). EEG was segmented from 800 ms before the stimulus onset to 2000 ms after the stimulus onset in each condition. The EEG was baseline-corrected to an 800 ms recording interval. Trials were discarded from analysis if there were voltage fluctuations of over 1,000 μV or data were deemed to be mathematically improbable, with this probability threshold set at 5 standard deviations.

Table 1 reports the average number of trials for each condition across subjects. Infomax-based independent component analysis (ICA) (Bell and Sejnowski, 1995) was run. Semi-Automated Selection of Independent Components of the electroencephalogram for Artifact correction (SASICA) (Chaumon et al., 2015), along with manual inspection, was used to identify and remove noise components (eye-blink and eye-movement artifact). There were one to three components rejected for each subject and session based on ICA. Data were converted to the time frequency domain using a Morlet wavelet transformation. Average spectral power changes relative to baseline in frontal and parietal channels were compared across conditions.

**TABLE 1:** Number of trials for each condition.

	**Experimental**		**Control**	
**Condition**	**Pre-training**	**Post-training**	**Pre-training**	**Post-training**
Place	73.70 (10.53)	69.85 (11.67)	71.25 (6.54)	65.90 (9.30)
Pleasantness	73.55 (7.82)	71.05 (12.24)	72.50 (7.82)	70.40 (11.60)
New	132.60 (32.23)	142.45 (23.09)	141.80 (30.39)	147.65 (27.15)
Place correct source	49.60 (7.89)	48.20 (12.05)	51.95 (9.62)	46.05 (8.17)
Place incorrect source	27.15 (8.62)	24.50 (8.54)	21.75 (10.35)	22.05 (8.34)
Pleasantness correct source	46.15 (12.68)	53.40 (14.17)	48.25 (18.53)	53.85 (15.91)
Pleasantness incorrect source	30.55 (11.17)	21.10 (7.50)	26.65 (15.85)	18.95 (10.22)

*Means with standard deviations in parentheses.*

#### Mindfulness Meditation Training

After the initial experimental session, the subjects in the mindfulness meditation experimental group underwent 4 weeks of mindfulness meditation training and practice. The group met with a professional meditation instructor for 1 h each week, in which the subjects were taught the techniques of practicing mindfulness meditation. The mindfulness meditation course incorporated aspects of the standard Mindfulness Based Stress Reduction (MBSR) course (Kabat-Zinn, 1990) such as a breath awareness and sitting meditation, which includes becoming aware of automatic or unintentional thoughts or feelings and letting these distractions pass.

Outside of the group training sessions, the subjects were instructed to utilize the skills learned in the course and practice mindfulness meditation on their own for at least 20 min each day using a guided breath awareness meditation recording. Therefore, throughout the 4-week training subjects were expected to practice mindfulness meditation a total of 720 min. Mindfulness meditation was tracked by the mindfulness meditation experimental group subjects responding to daily emailed surveys (Qualtrics, Provo, UT, United States) which asked how many minutes they practiced mindfulness meditation, what they did during their meditation, and how the practice was going for them thus far.

For the control group, pre-training and post-training were conducted while the subjects were on a waiting list for mindfulness meditation training. The randomly assigned waitlist control group controlled for non-specific factors such as practice effects from doing the episodic memory task twice and timing for the two experimental sessions.

## Results

Behavioral and EEG results were assessed using mixed analysis of variance (ANOVA) using SPSS version 24 software (IBM Corporation, Inc., Armonk, NY, United States). For each ANOVA we tested the normality and equality of variance. The only violations of equality of variance were in the behavioral results for source *c* and in the EEG results for source accuracy. In addition, all statistics report Greenhouse–Geisser correction for violations of sphericity. All *post hoc* tests corrected for multiple comparisons.

### Behavioral Results

#### Mindfulness Questionnaires

Based on self-reports, subjects spent an average of 399 min practicing mindfulness meditation over the course of the 4 weeks of mindfulness meditation training. Subjects spent an average of 20 min per day over an average of 20 days practicing mindfulness meditation. Therefore, on average subjects practiced mindfulness meditation about 55% of the time that they were expected to. Mindfulness was measured using the FFMQ. Data were missing for four subjects: two subjects were missing data for one question for the Observe scale, one subject was missing data for one question for the Describe scale, and one subject was missing data for one question for the Awareness scale of the FFMQ. These data were missing because subjects failed to respond to a question for these scales. Their data were replaced with the linear regression trend value for that point and used in all subsequent analyses.

FFMQ Total as well as for each factor (Observe, Describe, Awareness, Non-judge, and Non-reactive) were compared between mindfulness meditation experimental and waitlist control groups across time with separate Group (experimental, control) × Time (pre-training, post-training) mixed ANOVA. FFMQ Total scores were higher for the mindfulness meditation experimental than the waitlist control group [*F*(1,38) = 4.58, mean square error *(MSE)* = 497.27, *p* = 0.04]. FFMQ Total scores were higher post-training than pre-training [*F*(1,38) = 4.27, *MSE* = 67.67, *p* = 0.05]. Group interacted with time such that FFMQ Total scores were higher post-training than pre-training for the mindfulness meditation experimental but not the waitlist control group [*F*(1,38) = 11.15, *MSE* = 67.67, *p* < 0.01]. The difference between pre-training and post-training was significant only for the mindfulness meditation experimental group [*F*(1,19) = 15.60, *MSE* = 63.34, *p* < 0.01]. FFMQ Observe scores were higher for the mindfulness meditation experimental than the waitlist control group [*F*(1,38) = 7.77, *MSE* = 42.76, *p* < 0.01]. FFMQ Describe scores were higher for the mindfulness meditation experimental than the waitlist control group [*F*(1,38) = 6.15, *MSE* = 47.79, *p* = 0.02]. There was a marginal interaction between group and time such that FFMQ Describe scores were higher post-training than pre-training for the mindfulness meditation experimental but not the waitlist control group [*F*(1,38) = 3.35, *MSE* = 12.26, *p* = 0.08]. The difference between pre-training and post-training was significant only for the mindfulness meditation experimental group [*F*(1,19) = 6.36, *MSE* = 8.44, *p* = 0.02]. Group interacted with time such that FFMQ Awareness scores were higher post-training than pre-training for the mindfulness meditation experimental but not the waitlist control group [*F*(1,38) = 4.20, *MSE* = 10.12, *p* = 0.05]. There was a marginal interaction between group and time such that FFMQ Non-judge scores were higher post-training than pre-training for the mindfulness meditation experimental but not the waitlist control group [*F*(1,38) = 3.87, *MSE* = 15.37, *p* = 0.06]. The difference between pre-training and post-training was significant only for the mindfulness meditation experimental group [*F*(1,19) = 10.12, *MSE* = 8.60, *p* < 0.01] (see Table 2).

**TABLE 2:** Five facet mindfulness questionnaire data.

	**Experimental**		**Control**	
	**Pre-training**	**Post-training**	**Pre-training**	**Post-training**
Total	128.13 (2.38)	138.07 (3.24)	123.59 (4.19)	121.25 (4.77)
Observe	26.98 (1.16)	28.70 (1.00)	23.83 (1.14)	23.70 (1.26)
Describe	29.5 (1.36)	31.82 (0.99)	27.10 (1.25)	26.55 (1.26)
Awareness	25.25 (1.06)	26.95 (1.12)	25.27 (0.94)	24.05 (1.28)
Non-judge	24.65 (1.26)	27.60 (1.40)	27.50 (1.42)	27.00 (2.05)
Non-reactive	21.75 (0.99)	23.00 (1.08)	19.90 (1.09)	19.95 (1.16)

*Means with standard errors in parentheses.*

#### Episodic Memory

Recognition memory analyses were performed on item and source discrimination (*d*′) [*Z*(hit rate) – *Z*(false alarm rate)] and response bias (*c*) [−0.5(*Z*(hit rate) – *Z*(false alarm rate))]. Item *d*′ and *c* was measured independently from source *d*′ and *c* as previous studies have done for source memory (Murnane and Bayen, 1996; Slotnick and Dodson, 2005). For the place and the pleasantness task, an item hit was defined as a “Place” or “Pleasant” response to an old item, regardless of whether they classified the source correctly. Conversely, an item false alarm (FA) was a “Place” or “Pleasant” response to a new item. A source hit was anytime a subject responded “Place” for an item studied in the place task and a source FA was anytime a subject responded “Place” for an item studied in the pleasantness task. Item *d*′ and *c* were calculated by comparing old to new words for both the place and the pleasantness task (hit place task – FA and hit pleasantness task – FA) whereas source *d*′ was calculated for item hits only (hit place correct source – hit pleasant incorrect source). Assignment of the place vs. pleasantness tasks to hits vs. FAs for computing source *d*′ and *c* was completely arbitrary, and equivalent results would be obtained through the opposite assignment.

Item *d*′ and *c* were compared between experimental and waitlist control groups with a Group × Time × Task (place, pleasantness) mixed ANOVA. Item *d*′ was higher post-training than pre-training [*F*(1,38) = 4.19, *MSE* = 0.16, *p* = 0.05]. Item *d*′ was higher following the pleasantness task than the place task [*F*(1,38) = 11.16, *MSE* = 0.04, *p* < 0.01]. There was no main effect or interactions involving the group factor, including a group × time interaction [*F*(1,38) = 0.002, *MSE* = 0.16] or group × time × task interaction [*F*(1,38) = 0.02, *MSE* = 0.04]. Item *c* was higher post-training than pre-training [*F*(1,38) = 11.50, *MSE* = 0.08, *p* < 0.01]. Item *c* was higher following the place task than the pleasantness task [*F*(1,38) = 10.75, *MSE* = 0.01, *p* < 0.01] (see Table 3).

**TABLE 3:** Item behavioral data.

		**Experimental**		**Control**	
	**Condition**	**Pre-training**	**Post-training**	**Pre-training**	**Post-training**
Hit	Place	0.81 (0.03)	0.77 (0.03)	0.77 (0.01)	0.72 (0.02)
	Pleasantness	0.81 (0.02)	0.79 (0.03)	0.79 (0.02)	0.77 (0.03)
FA		0.25 (0.04)	0.19 (0.03)	0.22 (0.03)	0.17 (0.03)
Item *d*′	Place	1.68 (0.12)	1.77 (0.09)	1.64 (0.11)	1.72 (0.11)
	Pleasantness	1.71 (0.15)	1.88 (0.11)	1.74 (0.12)	1.92 (0.14)
Item *c*	Place	−0.08(0.09)	0.10 (0.10)	0.07 (0.08)	0.24 (0.09)
	Pleasantness	−0.09(0.08)	0.04 (0.09)	0.02 (0.08)	0.14 (0.09)

*Means with standard errors in parentheses.*

Source *d*′ and *c* were compared between experimental and waitlist control groups with a Group × Time mixed ANOVA. Source *d*′ was higher post-training than pre-training [*F*(1,38) = 12.80, *MSE* = 0.12, *p* < 0.01]. There was no main effect or interactions involving the group factor, including a group × time interaction [*F*(1,38) = 1.16, *MSE* = 0.12]. Although there was no main effect of group or interaction between group and time [*F*(1,38) = 1.16, *MSE* = 0.12], pairwise comparisons investigated potential differences that were predicted based on previous research and are apparent in the data. The difference between pre-training and post-training was significant only for the mindfulness meditation experimental group [*F*(1,19) = 10.53, *MSE* = 0.12, *p* < 0.01; control: *F*(1,19) = 3.22, *MSE* = 0.12, *p* = 0.09]. Source *c* was higher post-training than pre-training [*F*(1,38) = 11.15, *MSE* = 0.06, *p* < 0.01] (see Table 4).

**TABLE 4:** Source behavioral data.

		**Experimental**		**Control**	
	**Condition**	**Pre-training**	**Post-training**	**Pre-training**	**Post-training**
Hit	Place correct source	0.66 (0.02)	0.67 (0.03)	0.71 (0.03)	0.69 (0.02)
	Pleasantness correct source	0.61 (0.03)	0.72 (0.03)	0.64 (0.05)	0.74 (0.03)
FA	Place incorrect source	0.34 (0.02)	0.33 (0.03)	0.29 (0.03)	0.31 (0.02)
	Pleasantness incorrect source	0.39 (0.03)	0.28 (0.03)	0.36 (0.05)	0.26 (0.03)
Source *d*′		0.70 (0.11)	1.06 (0.12)	1.04 (0.17)	1.23 (0.14)
Source *c*		−0.06(0.05)	0.07 (0.05)	−0.12(0.12)	0.10 (0.07)

*Means with standard errors in parentheses.*

Reaction times (RTs) on only correct trials were analyzed first with a Group × Time × Memory Status (correct rejection of new words place, pleasantness) mixed ANOVA. RTs were faster for new words than words following the place and the pleasantness tasks [*F*(1.53,58.07) = 137.25, *MSE* = 13373, *p* < 0.01]. The difference between old and new words was significant following the place [*F*(1,39) = 134.36, *MSE* = 7945, *p* < 0.01] and the pleasantness task [*F*(1,39) = 178.52, *MSE* = 5803, *p* < 0.01]. Time interacted with memory status such that RTs were faster post-training than pre-training for new words and faster pre-training than post-training for words following the place and the pleasantness task [*F*(1.80,68.53) = 5.82, *MSE* = 3365, *p* < 0.01] (see Table 5).

**TABLE 5:** Item reaction time data.

		**Experimental**		**Control**	
	**Condition**	**Pre-training**	**Post-training**	**Pre-training**	**Post-training**
RT	Place	1517 (50)	1504 (42)	1543 (40)	1583 (32)
	Pleasantness	1504 (53)	1529 (44)	1524 (44)	1575 (34)
	New	1352 (62)	1285 (50)	1280 (41)	1306 (38)

*Means with standard errors in parentheses.*

Second, RTs on only item-correct old trials were analyzed with a Group × Time × Task × Source Accuracy (correct, incorrect) mixed ANOVA. RTs were faster for correct than incorrect source judgments [*F*(1,38) = 32.49, *MSE* = 15951, *p* < 0.01]. Task interacted with source accuracy such that the difference in RTs between correct and incorrect source judgments was greater following the place than the pleasantness task [*F*(1,38) = 4.22, *MSE* = 16846, *p* = 0.05]. The difference between correct and incorrect source judgments was significant only following the place task [*F*(1,39) = 39.62, *MSE* = 6142, *p* < 0.01]. There was an interaction between time, task, and source accuracy such that RTs were faster pre-training than post-training for correct source judgments following the place task and incorrect source judgments following the pleasantness task, and faster post-training than pre-training for incorrect source judgments following the place task [*F*(1,38) = 9.05, *MSE* = 9278, *p* < 0.01]. The difference between pre-training and post-training was significant only for incorrect source judgments following the pleasantness task [*F*(1,39) = 5.58, *MSE* = 20282, *p* = 0.02] (see Table 6).

**TABLE 6:** Source reaction time data.

		**Experimental**		**Control**	
	**Condition**	**Pre-training**	**Post-training**	**Pre-training**	**Post-training**
RT	Place correct source	1447 (52)	1471 (42)	1474 (39)	1534 (32)
	Place incorrect source	1586 (50)	1537 (47)	1611 (49)	1633 (40)
	Pleasantness correct source	1506 (54)	1499 (45)	1509 (39)	1518 (35)
	Pleasantness incorrect source	1502 (56)	1559 (51)	1540 (59)	1633 (41)

*Means with standard errors in parentheses.*

### EEG Results

**FIGURE 2:**
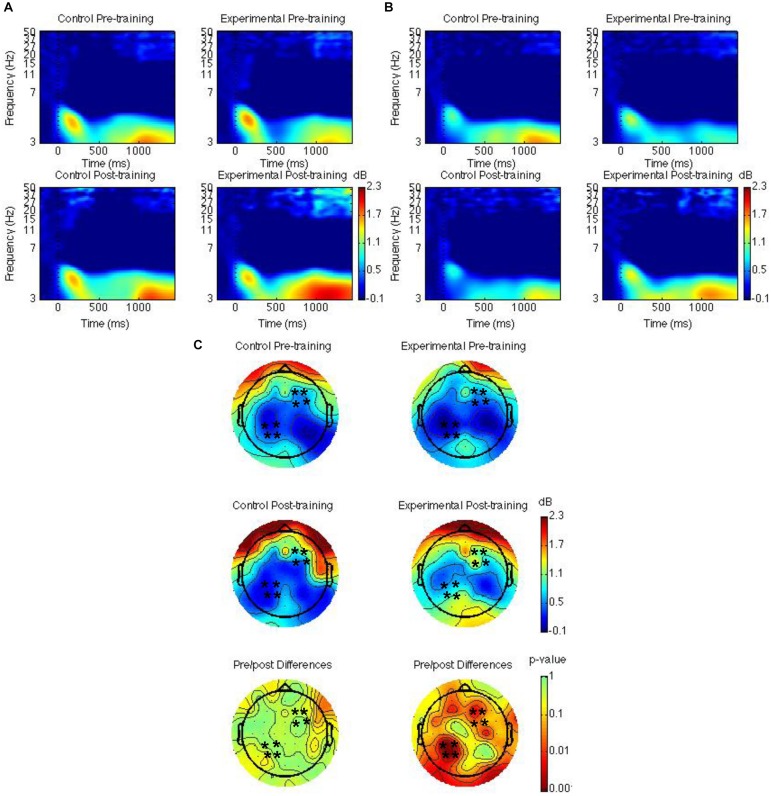
Theta power for the mindfulness meditation experimental and the waitlist control group for the pre-training compared to the post-training session. Time-frequency spectrograms across times and frequencies in a right frontal channel **(A)**. Time-frequency spectrograms across times and frequencies in a left parietal channel **(B)**. Theta power across all channels from 1000 to 1500 ms and differences from pre-training to post-training. Black ^∗^ marks analyzed channels in right frontal and left parietal regions **(C)**. Color scale: decibel change from pre-stimulus baseline and *p*-value of pre-training to post-training differences.

**FIGURE 3:**
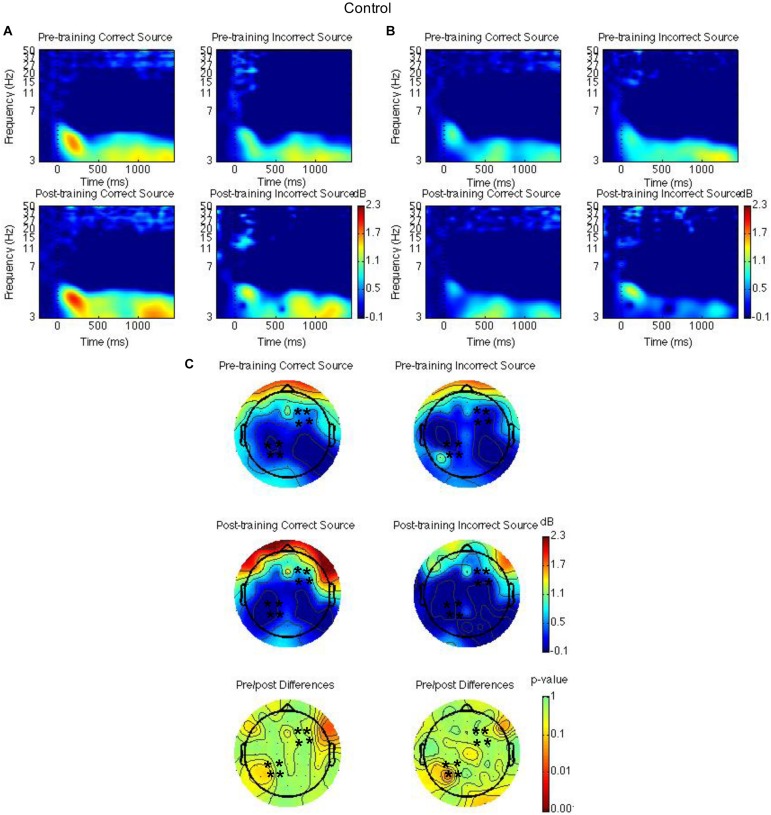
Theta power for the waitlist control group for the pre-training compared to the post-training session for hits with correct source and hits with incorrect source. Time-frequency spectrograms across times and frequencies in a right frontal channel **(A)**. Time-frequency spectrograms across times and frequencies in a left parietal channel **(B)**. Theta power across all channels from 1000 to 1500 ms and differences from pre-training to post-training. Black ^∗^ marks analyzed channels in right frontal and left parietal regions **(C)**. Color scale: decibel change from pre-stimulus baseline and *p*-value of pre-training to post-training differences.

**FIGURE 4:**
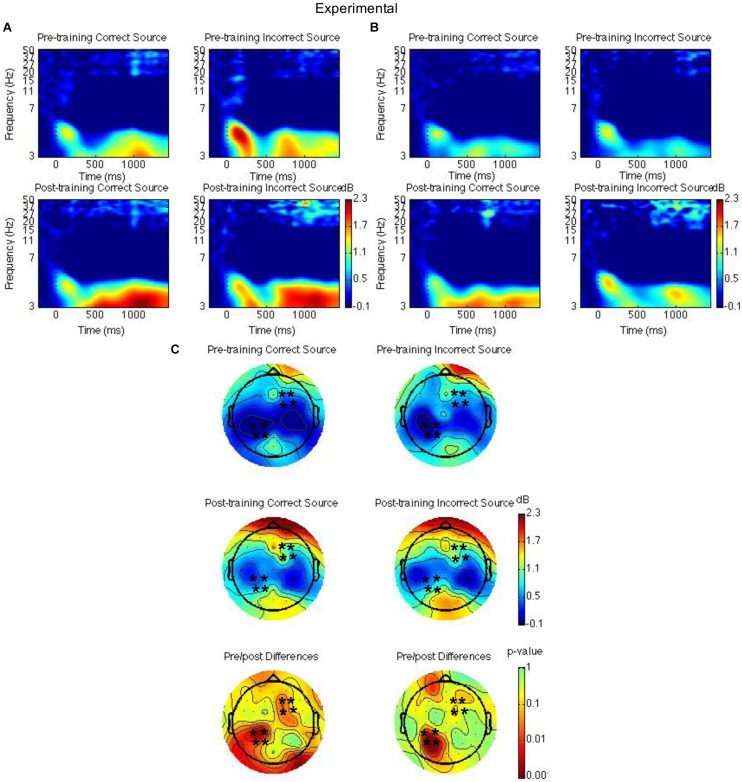
Theta power for the mindfulness meditation experimental group for the pre-training compared to the post-training session for hits with correct source and hits with incorrect source. Time-frequency spectrograms across times and frequencies in a right frontal channel **(A)**. Time-frequency spectrograms across times and frequencies in a left parietal channel **(B)**. Theta power across all channels from 1000 to 1500 ms and differences from pre-training to post-training. Black ^∗^ marks analyzed channels in right frontal and left parietal regions **(C)**. Color scale: decibel change from pre-stimulus baseline and *p*-value of pre-training to post-training differences.

Spatiotemporal regions of interest (ROIs) were defined according to previous research showing event-related potential (ERP) and theta oscillatory effects during source memory retrieval (Nyhus and Badre, 2015; Ross et al., 2015, 2018; Medrano et al., 2017). The ROIs were left parietal and right frontal channel groups (see Figures 2C, 3C, 4C); mean theta (4–8 Hz) power from 1000 to 1500 ms was computed by averaging the channels within each region for each condition/subject. Time-frequency spectrograms across times and frequencies in a right frontal and a left parietal channel, topographic plots of theta power across all channels from 1000 to 1500 ms, and differences from pre-training to post-training are shown in Figures 2–4. Right frontal and left parietal theta power on only correct trials were separately analyzed first with a Group × Time × Memory Status mixed ANOVA. For the right frontal channels and left parietal channels one outlier (theta power < 5 SD of the mean for more than one condition) in the control group was removed from analyses. Theta power in right frontal channels was greater post-training than pre-training [*F*(1,37) = 4.40, *MSE* = 1.55, *p* = 0.04]. Theta power was greater for new words than words following the place and the pleasantness tasks [*F*(2,73.95) = 4.43, *MSE* = 0.35, *p* = 0.02]. The difference between old and new words was significant only following the pleasantness task [*F*(1,38) = 8.24, *MSE* = 0.18, *p* < 0.01] (see Figures 2A,C). Theta power in left parietal channels was marginally greater post-training than pre-training [*F*(1,37) = 3.85, *MSE* = 0.92, *p* = 0.06]. Theta power was greater for new words than words following the place and the pleasantness tasks [*F*(1.62,59.79) = 7.80, *MSE* = 0.83, *p* < 0.01]. The difference between old and new words was significant following the place [*F*(1,38) = 9.66, *MSE* = 0.44, *p* < 0.01] and the pleasantness task [*F*(1,38) = 8.91, *MSE* = 0.39, *p* < 0.01]. Group interacted with time such that theta power was greater post-training than pre-training for the mindfulness meditation experimental but not the waitlist control group [*F*(1,37) = 9.52, *MSE* = 0.92, *p* < 0.01]. The difference between pre-training and post-training was significant only for the mindfulness meditation experimental group [*F*(1,19) = 17.37, *MSE* = 0.23, *p* < 0.01] (see Figures 2B,C).

Second, right frontal and left parietal theta power from 1000 to 1500 ms on only item-correct old trials were separately analyzed with a Group × Time × Task × Source Accuracy mixed ANOVA. For the right frontal channels, group interacted with time such that theta power was greater post-training than pre-training for the mindfulness meditation experimental but not the waitlist control group [*F*(1,37) = 5.28, *MSE* = 2.31, *p* = 0.03]. The difference between pre-training and post-training was significant only for the mindfulness meditation experimental group [*F*(1,19) = 10.15 *MSE* = 0.34, *p* < 0.01] (see Figures 3A,C, 4A,C). For the left parietal channels, group interacted with time such that theta power was greater post-training than pre-training for the mindfulness meditation experimental but not the waitlist control group [*F*(1,37) = 13.18, *MSE* = 2.18, *p* < 0.01]. The difference between pre-training and post-training was significant only for the mindfulness meditation experimental group [*F*(1,19) = 15.47 *MSE* = 0.31, *p* < 0.01]. There was a marginal three-way interaction between group, time, and source accuracy such that theta power was greater post-training than pre-training for both correct and incorrect source judgments for the mindfulness meditation experimental group but was greater pre-training than post-training for incorrect source judgments for the waitlist control group [*F*(1,37) = 3.43, *MSE* = 0.75, *p* = 0.07]. For the mindfulness meditation experimental group, the difference between pre-training and post-training was significant for correct [*F*(1,19) = 10.34, *MSE* = 0.42, *p* < 0.01] and incorrect source judgments [*F*(1,19) = 15.39, *MSE* = 0.30, *p* < 0.01] (see Figures 3B,C, 4B,C).

In addition, we examined EEG effects in other times (0–500, 500–1000, and 1000–1500 ms), other frequencies that have been related to mindfulness mediation or episodic memory, including alpha (8–12 Hz), beta (12–20 Hz), and gamma (25–50 Hz), and channels. The group × time effects were weaker than the theta power effects in right frontal and left parietal channels from 1000 to 1500 ms.

**FIGURE 5:**
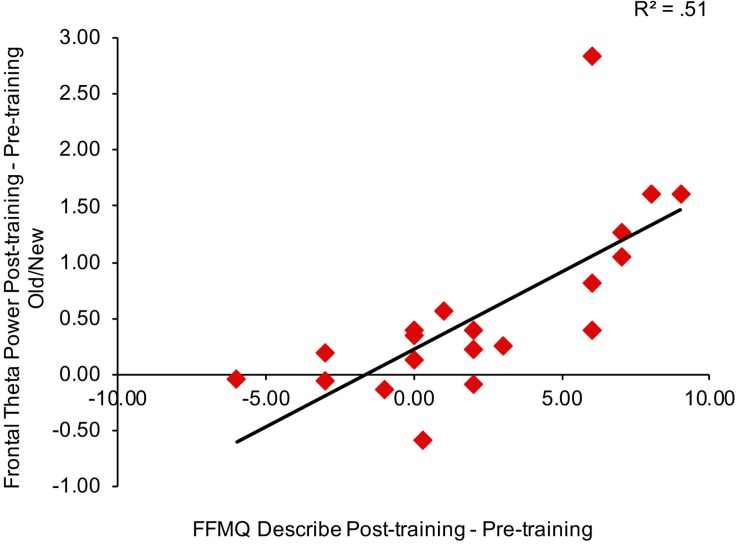
Correlation between the average difference in theta power between pre-training and post-training for hits and correct rejections in right frontal channels and the difference in FFMQ Describe scores between pre-training and post-training.

To consider the relationship between mindfulness meditation, source memory, and theta power effects, we examined the Pearson correlation between time spent practicing mindfulness meditation, FFMQ scores, memory performance (*d*′), and theta power in right frontal and left parietal channels for the mindfulness meditation experimental group. We focused on the variables that showed significant pre-training to post-training effects in the mindfulness meditation experimental group (FFMQ Total, Describe, and Non-judge, source *d*′, and theta power in right frontal and left parietal channels). In addition, we used the average difference in theta power between pre-training and post-training for hits and correct rejections and the average difference in theta power between pre-training and post-training for correct and incorrect source judgments because these conditions showed similar increases from pre-training to post-training. Specifically, the difference in source *d*′ and the average difference in theta power between pre-training and post-training for hits and correct rejections and the average difference in theta power between pre-training and post-training for correct and incorrect source judgments were correlated with time spent practicing mindfulness meditation and the difference in FFMQ Total, Describe, and Non-judge scores between pre-training and post-training. There were no outliers (>3 SD of the mean) used in the correlation analysis. There was a positive correlation between theta power pre/post old/new effects in right frontal channels and FFMQ Describe pre/post scores (*r* = 0.72, *n* = 20, *p* < 0.01, two-tailed, Bonferroni corrected) (see Figure 5).

We reanalyzed the correlations after removing the subjects with missing FFMQ scores (*n* = 2 in the mindfulness meditation experimental group). The pattern of results was similar for all subjects and subjects with missing FFMQ scores removed, there was a positive correlation between theta power pre/post old/new effects in right frontal channels and FFMQ Describe pre/post scores for subjects with missing FFMQ scores removed (*r* = 0.74, *n* = 18, *p* < 0.01, two-tailed, Bonferroni corrected).

## Discussion

The purpose of the present experiment was to determine if mindfulness meditation affects source memory and theta oscillations. Subjects spent a substantial amount of time practicing mindfulness meditation which led to increases in mindfulness as measured by the FFMQ. Source discrimination was greater post-training than pre-training for the mindfulness meditation experimental group. EEG results revealed that right frontal and left parietal theta power between 1000 and 1500 ms was greater post-training than pre-training for the mindfulness meditation experimental group. FFMQ Describe pre/post scores correlated with theta power pre/post old/new effects in right frontal channels.

Although subjects spent a substantial amount of time practicing mindfulness meditation throughout the 4-week training, they did not complete the full 8-week MBSR course and on average subjects practiced mindfulness meditation about 55% of the time that they were expected to. Despite this, subjects’ mindfulness increased from pre-training to post-training for the FFMQ Total, Describe, and Non-judge scales whereas waitlist control group FFMQ scores were similar from pre-training to post-training. Therefore, 4 weeks of mindfulness meditation training was sufficient to increase mindfulness.

Source discrimination was greater post-training than pre-training for the mindfulness meditation experimental group whereas for the waitlist control group source discrimination was similar from pre-training to post-training. These results should be interpreted with caution because they arose from pairwise comparisons that were not accompanied by a significant time by group interaction, but they are consistent with previous research showing effects of mindfulness meditation on recognition memory, especially recollection (Brown et al., 2016; Basso et al., 2019). These results are inconsistent with previous research showing detrimental effects of mindfulness meditation on episodic memory (reviewed in Levi and Rosenstreich, 2018). For example, Wilson et al. (2015) found increased false alarms in the Deese-Roediger-McDermott paradigm and in a recognition task following a short mindfulness induction indicating decreased monitoring following mindfulness meditation (Wilson et al., 2015). Whereas we trained participants in mindfulness meditation over the course of a month, Wilson et al. (2015) did a 15-min mindfulness induction prior to the memory task. Therefore, it is possible that training in mindfulness meditation causes long-lasting changes to brain structure and function that benefit memory, whereas the cognitive demands of a short mindfulness induction disrupt memory.

The EEG results are consistent with previous research showing greater theta power in both long-term meditators and following mindfulness meditation training (reviewed in Delmonte, 1984; Lou et al., 1999; Kubota et al., 2001; Aftanas and Golosheikin, 2003; reviewed in Cahn and Polich, 2006; Tang et al., 2009; reviewed in Fell et al., 2010; reviewed in Lomas et al., 2015; Brandmeyer and Delorme, 2018; reviewed in Lee et al., 2018). But theta power increases from pre-training to post-training were not specific to memory conditions. Therefore, these results suggest that following meditation training, there is a general increase in theta oscillations in brain regions related to episodic memory.

In addition, FFMQ Describe pre/post scores correlated with theta power pre/post old/new effects in right frontal channels. These results provide an important link between mindfulness meditation and theta oscillations during episodic memory retrieval and suggest that theta oscillations during episodic memory may be enhanced specifically by an increased ability to describe internal thoughts and feelings. Combined, the behavioral and EEG results suggest that increased mindfulness leads to better source memory and increased theta oscillations.

Mindfulness meditation may prove beneficial in aging and for patients suffering from mental illness that show disruption of oscillatory activity and memory impairment. Although some evidence suggests that mindfulness meditation has positive effects on cognition in aging and Alzheimer’s disease (reviewed in Marciniak et al., 2014) and may protect against cortical thinning with age (Lazar et al., 2005), it is not clear how these effects compare to other interventions that have also been shown to improve memory performance such as nutrition, exercise, and non-invasive brain stimulation [transcranial magnetic stimulation (TMS) and transcranial alternating current stimulation (tACS)] and change the structure and function of brain networks related to episodic memory.

### Limitations

Although the present results suggest that mindfulness meditation leads to better source memory, the interaction between group and time was not significant for source discrimination. This is likely due to both groups showing practice effects from doing the episodic memory task twice. In addition, this may have been due to the fact that the subject population was healthy young adults with good memory performance, therefore leaving little room for additional improvement. The mindfulness meditation course was only a month long, not the full 8-week MBSR course, and on average subjects’ daily practice was not as long as expected. Although subjects spent an average of 20 min per day over an average of 20 days practicing mindfulness meditation, some subjects spent as little as 15–16 min per day and other subjects spent as little as 6–7 days practicing mindfulness meditation, suggesting that it was difficult for subjects to maintain a daily practice. Therefore, the total time spent practicing mindfulness meditation was limited. Previous research showed that 8 weeks, but not 4 weeks of meditation led to improvement in recognition memory (Basso et al., 2019). Therefore, future research employing subjects with weaker memory using the full 8-week MBSR course may show stronger effects on source memory.

Although we used a randomized controlled design, there was no active control condition. Therefore, our results do not indicate whether mindfulness meditation alters memory function beyond other interventions. Future research should employ an active control condition that includes group training sessions and daily practice to determine effects specific to mindfulness meditation.

## Conclusion

Mindfulness meditation has been shown to improve episodic memory and increase theta oscillations, but no previous study has trained participants in mindfulness meditation and measured theta oscillatory effects during episodic memory. Combining mindfulness meditation training with behavioral and brain measures during episodic memory will enhance understanding of the neural processes affected by mindfulness meditation during episodic memory. Using a longitudinal design with matched mindfulness meditation and waitlist control groups and measuring EEG the present results indicate that mindfulness meditation increases activity in brain regions involved in the top-down control of memory retrieval as indexed by theta oscillations.

## Data Availability

The datasets generated for this study are available on request to the corresponding author.

## Ethics Statement

This study was carried out in accordance with the recommendations of the Department of Health and Human Services guidelines, Institutional Review Board of Bowdoin College with written informed consent from all subjects. All subjects gave written informed consent in accordance with the Declaration of Helsinki. The protocol was approved by the Institutional Review Board of Bowdoin College.

## Author Contributions

EN conceptualized the project, analyzed the behavioral and EEG data, interpreted the data, and wrote the manuscript. WE and TP collected the data. IV analyzed the EEG data.

## Conflict of Interest Statement

The authors declare that the research was conducted in the absence of any commercial or financial relationships that could be construed as a potential conflict of interest.
