# Supplementary material for: Increases in Theta Oscillatory Activity During Episodic Memory Retrieval Following Mindfulness Meditation Training
Source: Front Hum Neurosci. 2019 Sep 4;13:311. doi: 10.3389/fnhum.2019.00311 (PMC6738165; doi:10.3389/fnhum.2019.00311)
Supplement: Supplementary file 1 [file Data_Sheet_1.PDF]

Five Facet Mindfulness Questionnaire (FFMQ)
Ruth A. Baer, Ph.D.
University of Kentucky

Please rate each of the following statements using the scale provided. Write the number in the blank that best describes your own opinion of what is generally true for you.

1	2	3	4	5
never or very rarely true	rarely true	sometimes true	often true	very often or always true

_____ 1. When I'm walking, I deliberately notice the sensations of my body moving.

_____ 2. I'm good at finding words to describe my feelings.

_____ 3. I criticize myself for having irrational or inappropriate emotions.

_____ 4. I perceive my feelings and emotions without having to react to them.

_____ 5. When I do things, my mind wanders off and I'm easily distracted.

_____ 6. When I take a shower or bath, I stay alert to the sensations of water on my body.

_____ 7. I can easily put my beliefs, opinions, and expectations into words.

_____ 8. I don't pay attention to what I'm doing because I'm daydreaming, worrying, or otherwise distracted.

_____ 9. I watch my feelings without getting lost in them.

_____ 10. I tell myself I shouldn't be feeling the way I'm feeling.

_____ 11. I notice how foods and drinks affect my thoughts, bodily sensations, and emotions.

_____ 12. It's hard for me to find the words to describe what I'm thinking.

_____ 13. I am easily distracted.

_____ 14. I believe some of my thoughts are abnormal or bad and I shouldn't think that way.

_____ 15. I pay attention to sensations, such as the wind in my hair or sun on my face.

_____ 16. I have trouble thinking of the right words to express how I feel about things

_____ 17. I make judgments about whether my thoughts are good or bad.

_____ 18. I find it difficult to stay focused on what's happening in the present.

_____ 19. When I have distressing thoughts or images, I "step back" and am aware of the thought or image without getting taken over by it.

_____ 20. I pay attention to sounds, such as clocks ticking, birds chirping, or cars passing.

_____ 21. In difficult situations, I can pause without immediately reacting.

1	2	3	4	5
**never or very rarely true**	**rarely true**	**sometimes true**	**often true**	**very often or always true**

_____ 22. When I have a sensation in my body, it's difficult for me to describe it because I can't find the right words.

_____ 23. It seems I am "running on automatic" without much awareness of what I'm doing.

_____24. When I have distressing thoughts or images, I feel calm soon after.

_____ 25. I tell myself that I shouldn't be thinking the way I'm thinking.

_____ 26. I notice the smells and aromas of things.

_____ 27. Even when I'm feeling terribly upset, I can find a way to put it into words.

_____ 28. I rush through activities without being really attentive to them.

_____ 29. When I have distressing thoughts or images I am able just to notice them without reacting.

_____ 30. I think some of my emotions are bad or inappropriate and I shouldn't feel them.

_____ 31. I notice visual elements in art or nature, such as colors, shapes, textures, or patterns of light and shadow.

_____ 32. My natural tendency is to put my experiences into words.

_____ 33. When I have distressing thoughts or images, I just notice them and let them go.

_____ 34. I do jobs or tasks automatically without being aware of what I'm doing.

_____ 35. When I have distressing thoughts or images, I judge myself as good or bad, depending what the thought/image is about.

_____ 36. I pay attention to how my emotions affect my thoughts and behavior.

_____ 37. I can usually describe how I feel at the moment in considerable detail.

_____ 38. I find myself doing things without paying attention.

_____ 39. I disapprove of myself when I have irrational ideas.

FFMQ Scoring instructions

For all items marked “R” the scoring must be reversed. Change 1 to 5, 2 to 4, 4 to 2, and 5 to 1 (3 stays unchanged). Then sum the scores for each subscale.

Observing

1, 6, 11, 15, 20, 26, 31, 36

Describing

2, 7, 12R, 16R, 22R, 27, 32, 37

Acting with awareness

5R, 8R, 13R, 18R, 23R, 28R, 34R, 38R

Nonjudging of inner experience

3R, 10R, 14R, 17R, 25R, 30R, 35R, 39R

Nonreactivity to inner experience

4, 9, 19, 21, 24, 29, 33
